# *Aedes* Mosquito Salivary Components and Their Effect on the Immune Response to Arboviruses

**DOI:** 10.3389/fcimb.2020.00407

**Published:** 2020-08-07

**Authors:** David Guerrero, Tineke Cantaert, Dorothée Missé

**Affiliations:** ^1^Immunology Unit, Institut Pasteur du Cambodge, Institut Pasteur International Network, Phnom Penh, Cambodia; ^2^MIVEGEC, IRD, University of Montpellier, CNRS, Montpellier, France

**Keywords:** arbovirus, mosquito saliva, immune response, saliva composition, *Aedes* spp

## Abstract

Vector-borne diseases are responsible for over a billion infections each year and nearly one million deaths. Mosquito-borne dengue virus, West Nile, Japanese encephalitis, Zika, Chikungunya, and Rift Valley Fever viruses constitute major public health problems in regions with high densities of arthropod vectors. During the initial step of the transmission cycle, vector, host, and virus converge at the bite site, where local immune cells interact with the vector's saliva. Hematophagous mosquito saliva is a mixture of bioactive components known to modulate vertebrate hemostasis, immunity, and inflammation during the insect's feeding process. The capacity of mosquito saliva to modulate the host immune response has been well-studied over the last few decades and has led to the consensus that the presence of saliva is linked to the enhancement of virus transmission, host susceptibility, disease progression, viremia levels, and mortality. We review some of the major aspects of the interactions between mosquito saliva and the host immune response that may be useful for future studies on the control of arboviruses.

## Introduction

Each year, more than one billion people are infected with vector-borne diseases and nearly one million die as a result. These diseases take a toll on the health and quality of life of the population and have been shown to increase poverty in vulnerable communities due to illness and disability (WHO, 2020). Arboviruses represent an important group of pathogens that cause vector-borne diseases worldwide. There are more than 100 arboviruses known to cause disease in humans and they all share the common characteristic of being transmitted by hematophagous mosquitoes or ticks (LaBeaud et al., 2011). Although many infections are asymptomatic or cause a mild transient fever, progression to more severe pathologies, such as hemorrhagic fever, encephalitis, central nervous system involvement, or arthritis, can occur (Huang et al., 2019). Among the various arboviruses that can infect humans, mosquito-borne dengue (DENV), West Nile (WNV), Japanese encephalitis (JEV), Zika (ZIKV), Chikungunya (CHIKV), and Rift Valley Fever (RVFV) viruses are responsible for major public health problems in regions with high densities of arthropod vectors (Rolin et al., 2013; Wilder-Smith et al., 2017). DENV, WNV, JEV, and ZIKV belong to the *Flavivirus* genus of the *Flaviviridae* family. Their single-stranded positive-sense RNA genomes are typically 11 kb and encode seven non-structural proteins and three structural proteins (C, M, and E) (Laureti et al., 2018). On the other hand, CHIKV and RVFV belong to the *Alphaviridae* and *Phlebovirus* genera, respectively. CHIKV has a single-stranded positive-sense RNA genome that encodes four non-structural and five structural proteins, whereas RVFV has a tripartite genome, consisting of three single-stranded negative-sense RNA segments that together code for structural and non-structural proteins (Hartman, 2017; Vu et al., 2017). The transmission of most of these viruses is urban/peri-urban in human-mosquito infection cycles (Weaver and Reisen, 2010). As a result, there is a high rate of transmission in densely-populated regions (Struchiner et al., 2015). The successful propagation of these viruses worldwide can be attributed to various factors, amongst the most relevant, the wide distribution of *Aedes* spp. in all continents (Wilder-Smith et al., 2017), human travel and migration (Gould et al., 2017), animal trade (Pfeffer and Dobler, 2010), the creation of ecosystems that favor human-vector interactions (Kilpatrick and Randolph, 2012), and climate change, which favors vector populations (Papa, 2019). Transmission is initiated when a vertebrate host is inoculated with the virus during the blood-feeding of an infected mosquito. During this initial step of the cycle, vector, host, and pathogen converge at the bite site, making this event important for understanding the immune interactions in the skin microenvironment. Once the mosquito locates a vertebrate host, it penetrates the epidermis and dermis with its proboscis, and probes the site until it finds a suitable vessel or hemorrhagic blood pool (Townson, 1993). A salivary mixture containing pharmacologically active compounds, known to modulate host inflammation, hemostasis, and immunity, is injected during probing and facilitates blood intake by the insect by preventing clotting and platelet aggregation and promoting vasodilation (Manning and Cantaert, 2019). It is now known that certain molecules present in the vector's saliva can affect the course of infection, the replication capacity of the virus, and the host immune response (Schneider and Higgs, 2008; Pingen et al., 2017; Vogt et al., 2018). Here, we have provided a comprehensive overview of the anti-arboviral immune response, as well as its modulation by mosquito saliva, within the environment of the skin. We conclude with a prospective appraisal of the development of vaccines.

## *Aedes* Mosquito Salivary Components

Hematophagous mosquito saliva is a mixture of bioactive components known to modulate vertebrate hemostasis, immunity, and inflammation during the insect's feeding process. A recent proteome study identified 1,208 proteins from female *Aedes aegypti* salivary glands using LC-MS/MS analysis (Dhawan et al., 2017). This protein profile was further classified based on molecular functions assigned using bioinformatics databases. Translation, metabolism, oxidation-reduction, and cellular organization were the processes with the highest representation after classification. Furthermore, among the proteins identified in the study, a group of 238 was not attributed with any molecular function. Nevertheless, 64 of these unclassified proteins were predicted to have a signal peptide cleavage site and thought to be secreted. Importantly, salivary factor expression can vary depending on whether the mosquito has fed (blood fed or not) and the infection status (Thangamani and Wikel, 2009; Bonizzoni et al., 2012). These aspects need to be considered in studies examining the effects of mosquito saliva in the infection of target cells with arboviruses. Indeed, several studies have been conducted with saliva from uninfected mosquitoes. The nature of secretory bioactive factors in mosquito saliva is not restricted to proteins alone. In a study conducted by the University of Texas Medical Branch, the authors extracted short non-coding RNAs from the saliva of *A. aegypti* and *Aedes albopictus* mosquitos, infected or not with CHIKV (Maharaj et al., 2015). The researchers found novel microRNAs (miRNAs) expressed only during infection, which they believed could play an important role in regulating the establishment of infection in the vertebrate host during blood feeding. The amount of documented evidence from various disease models, pathogens, and mosquito species has led to the consensus that the presence of saliva and/or mosquito feeding is connected with enhancement of virus transmission, host susceptibility, disease progression, viremia levels, and mortality (Schneider and Higgs, 2008). To date, certain salivary factors have been separately characterized and their immunological role in infection determined (Table 1). However, this list is very limited and many studies remain to be conducted in order to better characterize the function of salivary components. The effects of salivary proteins in the host immune response can also vary as a function of the concentration of salivary-gland extracts (SGE). In some studies, high concentrations have been reported to be immune-suppressive, whereas low concentrations have been associated with modulation of the immune response, which normally corresponds to the downregulation of Th1 cytokines and a shift toward a Th2 response (Schneider and Higgs, 2008).

**Table 1:** Summary of salivary factors and their known or suspected role in host immunity.

**Factor**	**Species**	**Mechanism**	**Effect**	**References**
*A. aegypti* venom allergen-1 (*Aa*VA-1)	*A. aegypti*	Binds to autophagy inhibitor LRPPRC. Promotes autophagy activation.	Enhances viral replication in DCs and macrophages.	Sun et al., 2020
Neutrophil stimulating factor 1 (NeSt1)	*A. aegypti*	Induces expression of chemokines (pro-IL-1β, CXCL2 and CCL2)	Enhances viral replication. Promotes the recruitment of macrophages and activation of neutrophils.	Hastings et al., 2019
LTRIN	*A. aegypti*	Inhibits activation of NF-kB	Decreases the expression of pro-inflammatory cytokines	Jin et al., 2018
Anticlotting serpin-like protein (AT)	*A. aegypti*	Unknown	Enhances viral replication	Surasombatpattana et al., 2014
Adenosine deaminase (AD)	*A. aegypti*	Inhibits IFN-α and IFN-β mRNA expression	Enhances viral replication	
Putative 34 kDa family secreted salivary protein	*A. aegypti*	Inhibits type I IFN expression	Enhances viral replication	
Putative secreted protein (VA)	*A. aegypti*	Inhibits IFN-α and IFN-β mRNA expression	Enhances viral replication	
Serine protease CLIPA3	*A. aegypti*	Unknown	Enhances infection	Conway et al., 2014
*A. aegypti* bacteria-responsive protein 1 (AgBR1)	*A. aegypti*	Upregulates chemo-attractive chemokines, promotes neutrophil recruitment to bite site.	Enhances infection Increased disease severity	Uraki et al., 2019
miRNA-100	*A. aegypti*	Not known	Possible effect on the regulation of immune cell activity and influences viral replication.	Maharaj et al., 2015
miRNA-125	*A. aegypti*	Not known		

## Mosquito Bites and the Effect of Saliva on Viral Replication and Dissemination

The comparison between virus infection dynamics in animals infected through various inoculation routes, has permitted to observe the impact that mosquito-aided viral entry can have on the host. Mice infected with WNV via mosquito bite show higher viral loads and earlier dissemination from local inoculation sites to neighboring tissues than mice infected by needle injection, including earlier breach of the nervous system (Styer et al., 2011). In rhesus macaques, inoculation of DENV through infected mosquito feeding leads to higher and longer viremia than inoculation via subcutaneous, intradermal, or intradermal + SGE needle injection. In addition, macaques infected via mosquito feeding and intradermal + SGE injection show skin inflammation and cellular infiltration at the site of inoculation and higher levels of liver aminotransferase than those infected through other inoculation routes (McCracken et al., 2020). In a similar study, investigators found that macaques infected with ZIKV via mosquito bite developed systemic infection and altered tissue tropism to the virus, which was disseminated mainly in the hemolymphatic tissues, female reproductive tract, liver, and kidneys, whereas the virus was also detected in the cerebrum of one animal and the eyes of the two animals inoculated via subcutaneous needle injection (Dudley et al., 2017). No significant difference was observed in the peak viral load between the two groups, but the time to reach the viral load was different, with the subcutaneous group reaching the viremia peak faster than the mosquito-infected group. In the case of RVFV, intradermal infection of mice in conjunction with SGE leads to a significant increase in viral titers in blood, brain, and liver, and more severe thrombocyto/leukopenia (Le Coupanec et al., 2013). Additionally, mice intradermally infected with RVFV and exposed to non-infected mosquito bites show shorter survival. Similarly, a mouse model for infection using an avirulent strain of the Semliki Forest virus, a virus closely related to CHIKV, showed that mice exposed to the virus after *A. aegypti* feeding showed higher viral RNA levels at the inoculation site than unbitten mice, as well as earlier dissemination to the brain and evolution to a lethal outcome in some of the mice (Pingen et al., 2016). Although not explored exhaustively, the effect that varying the localization of the saliva inoculum induces in the host has also been investigated. Mice that receive WNV and SGE inoculated together show significantly higher viral titers than those that receive the virus and SGE separately in distal locations, highlighting the local effect of salivary factors on enhancing viremia (Styer et al., 2011).

## Effect of Saliva on Arbovirus Interactions with The skin Environment

**Figure 1:**
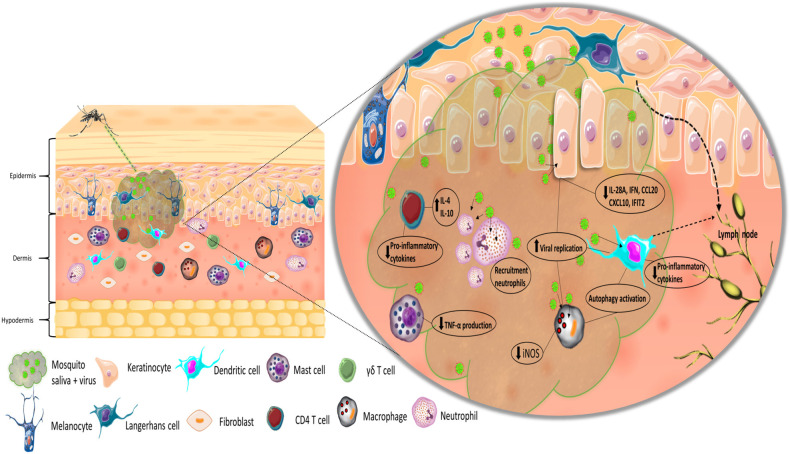
Simplified representation of the inoculation of virus and mosquito saliva into the skin. Recognition of the virus by LCs and DCs, and migration to lymph node. Effect of mosquito saliva on skin immune resident and infiltrating cells.

The first cells to interact with the virus are those that constitute the immune system within the skin, as the virus is directly injected into the epidermis and dermis of the host (Figure 1). Resident immune skin cells, such as Langerhans cells (LCs), occupy the epidermis together with keratinocytes, whereas various subpopulations of dendritic cells (DCs), macrophages, and T cells reside in the dermis (Matejuk, 2018), all of which can be targets of infection and sites of initial replication for arboviruses (Wu et al., 2000; Limon-Flores et al., 2005; Durbin et al., 2008; Silveira et al., 2018). Since these cells are both permissive to infection and hold the capacity to elicit immune responses, they play a dual role in the infection process: both as replication targets/dissemination vehicles and as the first line of defense. Keratinocytes are able to recognize pathogen-associated molecular patterns (PAMPs) through pattern recognition receptors (PRRs). Upon stimulation, they promote pro-inflammatory responses, along with the production of interferons (IFNs), chemokines, and cytokines (Miller, 2008; Briant et al., 2014). In a recent study, Garcia et al. (2018) observed that keratinocytes produce a type I and III interferon inflammatory response when infected with WNV that is associated with viral load. In addition, the expression of pro-inflammatory cytokines, such as TNF-α and IL-6, and various chemokines by keratinocytes increased significantly after viral infection. In the context of ZIKV, *in vitro* infection of skin fibroblasts leads to active viral replication of the virus (Hamel et al., 2015). Recognition of the virus by skin fibroblast is mostly mediated by RIG-I, MDA5, and TLR3 and induces IFN-α and IFN-β production, as well as that of the CXCR3 ligand CXCL10 and the antiviral chemokine CCL5. Fibroblasts have been reported to be highly susceptible to CHIKV and WNV infection (Ekchariyawat et al., 2015). Activation of these cells following infection appears to elicit similar antiviral responses to both viruses, which consist of increased levels of IL-1β (due to maturation of caspase 1), IFN-β, and other pro-inflammatory cytokines and chemokines. In this case, inflammasome activation via caspase 1 appears to be of particular importance in controlling CHIKV replication in fibroblasts. Keratinocytes infected with WNV in the presence of *A. aegypti* saliva show higher viral replication levels than cells infected without saliva at 48 h post-infection. Saliva-treated cells also present lower levels of mRNA for the inflammatory mediators IL-28A, CXCL10, IFIT2, and CCL20 24 h post-infection. Interestingly, treatment of infected keratinocytes with *Culex quinquefasciatus* saliva also leads to decreased levels of inflammatory mediators, but not to higher viral replication (Garcia et al., 2018). Similarly, keratinocytes show significantly higher viral loads at 6 and 24 h post-infection upon infection with DENV in the presence of *A. aegypti* saliva than those infected with virus alone. Decreased expression levels of β-defensin 3, LL-37, Elafin, and S100A7 at 6 h post-infection and IFNs at 24 h post-infection have also been observed (Surasombatpattana et al., 2012). *A. aegypti* bacteria-responsive protein 1 (AgBR1) is a protein upregulated in the salivary glands of mosquitoes after blood feeding (Ribeiro et al., 2007) that promotes the recruitment of CD45^+^CD11b^+^Ly6G^+^ cells, along with the upregulation of neutrophil- and monocytic-attracting chemokine expression at the bite site of mice infected with ZIKV via mosquito bite (Uraki et al., 2019). By stimulating the recruitment of cells susceptible to infection and dampening pro-inflammatory responses, mosquito salivary factors generate an auspicious environment for viral replication. Furthermore, as the earliest interactions of the immune system with arboviruses occur at the inoculation site, modulation of these processes give the virus an advantage in the infection-immune response dynamics.

## Effect of Saliva on the Innate Immune Response to Arboviruses

The modulatory effects of vector saliva factors on the innate immune system are an important contributing factor in the enhancement of viral replication and dissemination. In a recent study, Sun et al. identified a protein, designated *Aa*VA-1, that induces enhancement of viral replication of both ZIKV and DENV in DCs and macrophages (Sun et al., 2020). Silencing of this protein in the mosquitoes led to decreased viremia in ZIKV-infected mice, along with delayed death. Incubation of THP-1 cells, a monocytic cell line, with *Aa*VA-1 did not influence the production of TNF-α, IL-1β, IL-8, monocyte chemotactic protein-1 (MCP-1), IFN-β, IFN-α2, or IFN-α7. The investigators postulated that the protein enhances viral replication by a mechanism other than the modulation of Th1 cytokine production. Interestingly, use of an immuno-pulldown assay to identify proteins that bind to *Aa*VA-1 showed that the protein binds to the autophagy inhibitor, leucine-rich pentatricopeptide repeat-containing protein (LRPPRC). This inhibitor suppresses the initiation of autophagy by binding and sequestering Beclin-1. The ability of *Aa*VA-1 to bind to LRPPRC and displace Beclin-1, by competing for the same binding motif, enables the initiation of autophagy signaling and thus enhances viral transmission and replication. The effect of this salivary protein could therefore have a major impact on the severity of the disease. It is important to note however that ZIKV, like other flaviviruses, has developed sophisticated mechanisms to overcome autophagy (Chiramel and Best, 2018). Moreover, it has been reported that mosquito SGE significantly suppresses inducible nitric oxide synthase (iNOS) in macrophages. Suppressing the expression of iNOS lowers the levels of the reactive oxygen intermediate NO which is an important factor that regulates the functional activity, growth, and death of macrophages, T cells, NK cells, and neutrophils. Thus, the decrease of NO levels by mosquito saliva deregulates the activity of these immune cells (Schneider et al., 2010; Barros et al., 2019).

Neutrophils are an abundant cell population of the innate immune system. They are effective in controlling pathogens by degranulation, phagocytosis, and the formation of neutrophil extracellular traps (NETs) (Rosales, 2018). Murine neutrophils deploy NETs upon CHIKV infection, in a TLR7- and reactive oxygen species-dependent manner, which can neutralize the virus and lower its infective capacity (Hiroki et al., 2020). Furthermore, mice treated with DNase, which impairs the functionality of NETs, showed increased viral loads and were more susceptible to infection than controls. Indeed, the effect of neutrophils on disease protection can vary depending on the virus and the degree of NETs production (Opasawatchai et al., 2019). Neutrophil stimulating factor 1 (NeSt1) is a newly described salivary protein that enhances ZIKV replication and pathogenesis in mice (Hastings et al., 2019). The protein induces expression of pro-IL-1β, CXCL2, and CCL2, leading to the activation of primary neutrophils and further recruitment of macrophages, which are highly susceptible to infection. Interestingly, passive immunization against the NeSt1 protein prevents early replication and ameliorates pathogenesis in infected mice.

IFNs limit infection by inducing the expression of IFN-stimulated genes (ISGs), disrupting viral replication cycles at various steps (Sen, 2001). PRRs, such as RIG-1 and MDA5, can recognize viral genome particles in the cell cytosol (Hollidge et al., 2011) and their activation leads to type I IFN expression (Sharma, 2003). Other receptors, such as TLR3, TLR7, TLR8, and TLR9, promote type I IFN and NF-kB signaling pathway activation (Yamamoto et al., 2002; Kawai and Akira, 2007). The effectiveness of IFN responses to control arbovirus infections has been documented in several studies. In a hospital-based study in Taiwan, researchers reported that the serum of patients with severe dengue contained less IFN-α than that of patients with non-severe manifestations, supporting the relationship between IFN and protection (Chen et al., 2007). Jiang et al. observed that five ISGs were able to significantly inhibit both WNV and DENV replication and viral entry (Jiang et al., 2010). Additionally, the screening of more than 380 human genes using an overexpression-based approach identified several additional ISGs that suppress WNV replication (Schoggins et al., 2011). In the context of RVFV infection, it has been reported that a critical component of the host immune response to the virus is a robust type I IFN response shortly after infection (do Valle et al., 2010). In addition, early studies performed in rhesus macaques showed that macaques given IFN-α replacement therapy were able to control infection with RVFV (Morrill et al., 1989). In ZIKV *in vitro* studies, treatment of vaginal and cervical epithelial cells, important in the sexual transmission of Zika, with IFN-α/β or IFN-λ inhibited viral infection (Caine et al., 2019). In mouse models, CD4^+^ T cells, which have the ability to reduce the viral load in the brain, control disease progression, and prevent fatal outcome after ZIKV infection, appeared to be dependent on IFN-γ signaling pathways (Lucas et al., 2018). Additionally, infection of mice lacking IRF-3 and IRF-7 with CHIKV has been shown to lead to fatal outcomes due to hemorrhagic shock (Rudd et al., 2012; Schilte et al., 2012). These studies show the fundamental role of IFN responses in limiting arbovirus infections.

Studies from the early 90's already reported the inhibition of TNF-α production by rat mast cells cultured with SGE from *A. aegypt*i (Bissonnette et al., 1993) and the suppression of IL-2 and IFN-γ production by naïve spleen cells cultured with *A. aegypt*i SGE in mouse models (Cross et al., 1994). The downregulation of cellular pro-inflammatory responses affects viral infection and disease progression, as this mechanism is crucial for controlling viral infections. Polarization toward Th2 responses has been observed in mice after concomitant infection with the virus and salivary proteins. Mice that were exposed to *Culex pipiens* and *A. aegypti* for 4–10 days showed marked downregulation of IFN-γ production after mosquito feeding, along with upregulated production of IL-4 and IL-10 (Zeidner et al., 1999). In a separate study, cytokine expression in the skin of mice was analyzed after intradermal inoculation with *A. aegypti* SGE in combination with Sindbis virus (Schneider et al., 2004). Co-inoculation with the virus and SGE led to reduced production of IFN-β and IFN-γ. In contrast, the expression of IL-4 and IL-10 was significantly upregulated (Schneider et al., 2004). Upregulation of IL-10 production in mice has been associated with impaired Th1 cell responses, thus promoting prolonged and persistent viral infection (Brooks et al., 2006). In addition, humanized mice infected with DENV via mosquito bite show higher viremia than mice infected by injection (Cox et al., 2012). SGE itself appears to enhance thrombocytopenia in these animals.

An *in vitro* study performed on HaCaT and A549 cells showed dampened IL-8 production by cells treated with either CHIKV + SGE or SGE alone, alongside increased viral replication (Puiprom et al., 2013). Additionally, a comparative analysis of cytokine gene expression of mice bitten by uninfected mosquitoes and those bitten by mosquitoes infected with CHIKV showed an altered cytokine expression profile in both cases, characterized by the upregulation of IL-4 and downregulation of IL-2, IFN-γ, and TLR-3 relative to unbitten controls (Thangamani et al., 2010). The investigators also infected mice with CHIKV by needle intradermal injection and observed expression profiles characteristic of a pro-inflammatory response, increased IL-2, IFN-γ, and TLR-3 expression, along with decreased IL-4 and IL-10 expression, contrary to those observed for the mosquito-bitten cases. Interestingly, although the observed cytokine expression profiles of both the mice bitten by uninfected mosquitoes and those bitten by CHIKV-infected mosquitoes were similar and showed a shift from Th1 to Th2 responses, the effect on IL-4 expression appeared to be greater in the individuals bitten by CHIKV-infected mosquitoes. In a previous study, Thangamani et al. described similar effects of mosquito saliva/bites on the production of IL-4 using a TCR transgenic mouse model (Boppana et al., 2009). Here, exposure to both SGE and mosquito bite induced increased IL-4 expression by CD4^+^ T cells. Furthermore, the investigators were able to identify a newly described protein, salivary *A. aegypti* IL-4-inducing protein (SAAG-4), which upregulates IL-4 and IL-10 expression and downregulates the expression of IL-12, IFN-γ, and TNF-α in skin. The processes by which salivary proteins modulate cytokine production are not completely understood, but insights about possible mechanisms have become available in recent years.

In a recent study, researchers identified and characterized a 15-kD protein, LTRIN, isolated from *A. aegypti* salivary grands, which was found to be upregulated in blood-fed mosquitos (Jin et al., 2018). The investigators assessed the protein's immunosuppressive activity by infecting human THP-1 cells, human umbilical-vein endothelial cells, mouse BMDMs, and mouse skin fibroblasts with ZIKV in the presence or absence of the protein. In the presence of LTRIN, the level of intracellular transcripts of the virus increased in a dose-dependent manner in all cell types tested. Furthermore, LTRIN binds to the lymphotoxin-β receptor (LTβR), which initiates signaling pathways that regulate various processes, such as cell differentiation, development, and homeostasis of lymph nodes, type I IFN production, and hepatic regeneration (Ware, 2005; Gommerman et al., 2014). Binding of LTRIN to LTβR inhibits the dimerization and activation of the latter, resulting in the inhibition of the NF-κB signaling pathway and the subsequent production of pro-inflammatory cytokines (Jin et al., 2018). This is in accordance with the observations of the gene expression profile of the pro-inflammatory cytokines IL-1, IL-6, and TNF-α, which were expressed at a significantly lower level in cells treated with LTRIN. In a different study, four new proteins that are abundant in mosquito saliva were found to enhance the infection of human keratinocytes by DENV (Surasombatpattana et al., 2014). The proteins were identified after genomic and proteomic analyses of female *A. aegypti* salivary glands and shown to be an anticlotting serpin-like protein (AT), adenosine deaminase (AD), a putative 34-kDa family secreted salivary protein, and a putative secreted protein (VA). Higher DENV mRNA transcripts were found in keratinocytes incubated in the presence of each of these proteins than those incubated with the virus alone. Furthermore, of the four proteins, VA and AD were found to significantly inhibit IFN-α and IFN-β mRNA expression. The 34-kDa protein was reported to completely inhibit type I IFN expression. In accordance with its ability to inhibit type I IFN expression, the 34-kDa protein also inhibited the expression of both IRF3 and IRF7. Despite these advances, additional studies are still needed to determine the individual role that most of the salivary proteins of *A. aegypti* could play in the innate immune response against arboviruses. At present, the lack of these studies could be explained by the difficulty of producing recombinant salivary proteins. Another mechanism that enables *A. aegypti* saliva to modulate interferon responses involves interference of the Janus kinase/signal transducers and activators of transcription (JAK/STAT) signaling pathway. Activation of this pathway leads to the expression of ISG and many antiviral effectors (Nan et al., 2017) and in a recent study, it was observed to be affected by mosquito saliva (Wichit et al., 2017). The study reported downregulation of STAT2 and its phosphorylated form in human skin fibroblast infected with CHIKV in the presence of *A. aegypti* saliva, which led to a marked decrease in type I ISG production.

## The Effect of Saliva on the Complement System Response to Arbovirus

The complement system is a collection of more than 30 soluble and cell-surface proteins that recognize PAMPs. It can be activated by three different pathways: the classical pathway (CP), the lectin pathway (LP), and the alternative pathway (AP) (Stoermer and Morrison, 2011). The involvement of complement as a protective mechanism against arboviruses has been reported in various studies. Mice deficient for C3 or complement receptors 1 and 2 experience a higher WNV burden and higher mortality than wildtype mice (Mehlhop et al., 2005). In addition, in the context of WNV, mice genetically deficient for C1q (CP), C4 (CP, LP), factor B (AP), or factor D (AP) show higher mortality rates than wildtype mice. Additionally, brain infection was observed earlier for mice deficient for any one of these complement factors (Mehlhop and Diamond, 2006). Interestingly, the effects of complement on the dynamics of arbovirus infection may also operate outside of the human host. This is because complement factors can remain active after a blood meal and interact with the virus and cells within the mosquito, which can decrease viral loads in the vector and affect viral transmission (Londono-Renteria et al., 2016). Previous studies have demonstrated the capacity of Anopheline mosquito saliva to inhibit complement activity. SGE from *Anopheles albimanus, Anopheles freeborni*, and *Anopheles aquasalis*, all vectors for malaria, have been reported to inhibit the alternative pathway of the complement system (Mendes-Sousa et al., 2016, 2018). Nevertheless, studies carried out with *A. aegypti* saliva are consistent in demonstrating no direct inhibition of the complement system (Cavalcante et al., 2003; Pereira-Filho et al., 2020).

## The Effect of Saliva on the Lymphocyte Response to Arbovirus

T- and B-cell involvement in the response to arbovirus infection has also been shown to be highly important. CD4^+^ and CD8^+^ T cells differentiate into more specific subsets defined by different effector functions and cytokine profiles (Golubovskaya and Wu, 2016). The coordinated action of T and B cells leads to the production of specific antibodies against viral proteins. Mice depleted of CD4^+^ T cells and infected with attenuated RVFV, which is normally not able to generate disease, develop encephalitis, associated with lower virus-specific humoral and T-cell memory responses (Harmon et al., 2018). When mice are depleted of both CD4^+^ and CD8^+^ T cells, the incidence of encephalitis increases. Knockout mice unable to produce γδ T cells infected with CHIKV develop a more severe manifestation of the disease than wildtype controls (Long and Heise, 2015). These mice show increased inflammation-mediated oxidative damage in the feet and ankles and cytokine and chemokine production different from that of wildtype mice. In addition, infection of α/β-T cell deficient mice with JEV results in death over a 10- to 18-day period, in contrast to wildtype mice, which can survive infection, attributable to the granular lytic function of the cells (Jain et al., 2017). For DENV, lower percentages of regulatory B-cell subsets are associated with a more severe disease outcome during acute infection in humans (Upasani et al., 2019). Mosquito salivary factors have an effect on B- and T-cell responses and the proportion of these cell populations. Peripheral blood mononuclear cells treated with 2 μg of *A. aegypti* saliva show a higher proportion of NK T cells than untreated controls (Vogt et al., 2018). In this study, the authors observed no difference in the proportion of activated cells. Thus, the specific influence that this effect could have on the immune response to viral infections is difficult to assess. In the same study, Vogt et al. (2018) used humanized mice exposed to *A. aegypti* saliva through mosquito feeding and observed a lower number of IL-2-producing CD4^+^ T cells in the blood, and B cells in the skin 12 h after infection. Fewer CD4^+^ T cells in the blood were also observed at 48 h after infection. In this study, the populations of lymphocytes, NK cells, and other myeloid cells were analyzed at various time points, together with cytokine production, and the overall results are consistent with a mixed Th1/Th2 response, rather than the usual Th1 Th2 shift reported in many other papers. Cell death, together with decreased proliferation rates, is observed when mouse T cells are exposed to *A. aegypti* SGE. This suppressive effect is accompanied by the downregulation of Th1 and Th2 cytokine production (Wanasen et al., 2004; Bizzarro et al., 2013). In addition, B-cell proliferation is inhibited by *A. aegypti* SGE (Wasserman et al., 2004). Lymphocytes cultured in the presence of SGE showed alterations in the cell phenotype and an inversely proportional relationship between the number of viable cells and SGE concentration. The labeling of phosphatidylserine at the cell surface with annexin 5 confirmed the apoptosis of T and B lymphocytes. Lower levels of pro-caspase-3 and pro-caspase-8 expression in total spleen cell lysates than in controls was observed. This suggests that *A. aegypti* SGE mediates apoptosis in T and B lymphocytes via a caspase-3- and caspase-8-dependent pathway (Bizzarro et al., 2013).

## Vaccine Prospect

In the context of arbovirus, vaccine development has seen several breakthroughs in recent years and vaccines for JEV and yellow fever virus are licensed and used worldwide. Given the extensive diversity of arboviruses and their complex immunological interactions with the host, it is important to consider a variety of approaches to vaccine development. New ideas incorporate vector-based immunological concepts and have become a highly promising area of study. One such new vaccine strategy concerns the delivery of vaccines by microscale needles, which, in additional to being painless, more accurately mimic the natural inoculation of the virus by the arthropod vector and thus might elicit better immune activation in the skin (Manning and Cantaert, 2019). In 2018, researchers described the design of a microneedle based on the biology of mosquito mouth parts and the mechanism behind the painless bite, providing an example of innovative and better-designed vaccine delivery systems (Gurera et al., 2018). In comparison to traditional hypodermic needles, which deliver the vaccine into the subcutaneous strata of the skin, transcutaneous microneedle delivery appears to provide the advantage of promoting robust protective skin-resident responses. Furthermore, the painless and non-invasive nature of microneedle delivery systems, along with the minimal technical training required for their use, are highly desirable traits for a vaccine (Huang et al., 2019). In addition, the viral replication kinetics and evolution to peak viremia in the peripheral blood of macaques infected with DENV via mosquito bite are closer to the data reported for humans than those observed after subcutaneous delivery. Furthermore, the differences in replication kinetics that are observed between infection via mosquito bite and intradermal DENV inoculation do not disappear after the addition of SGE to the intradermal injection (McCracken et al., 2020). These results suggest that delivery systems and localization of the viral inoculum in the skin play an important role in determining viral replication kinetics. The use of salivary components as vaccine targets is also a concept that has gained interest over the years. If the vaccine can elicit an immune response at the very initial step of infection when host, vector, and virus interact, and if that response is triggered by the components present in the mosquito saliva, independent of the virus, then such a vaccine would boost the normal anti-viral response by creating an “anticipated” hostile environment for the virus-saliva mixture. This approach has the added advantage of being vector specific rather than virus specific, possibly opening the door to a catch-all vaccine for different viruses transmitted by a single vector (Manning and Cantaert, 2019). This concept also appears to be promising in the context of other non-viral arthropod-borne diseases. In hamster models, immunization against salivary protein LJM19 from *Lutzomyia longipalpis* provides protection against a fatal outcome from visceral leishmaniasis (Gomes et al., 2008). Also, immunity to salivary components has been documented in various studies with mammalian species after frequent exposure to ticks. This observations, together with studies on the effect of tick salivary proteins on the host immune response, has led to the identification of possible saliva vaccine candidates for tick-transmitted diseases (Manning et al., 2018). In addition, in the context of malaria, mice immunized with antiserum against an *Anopheles gambiae* salivary protein (TRIO) showed partial protection against plasmodium infection (Dragovic et al., 2018). In humans, the results from the first safety and immunogenicity phase 1 clinical trial for an universal mosquito-borne disease vaccine (AGS-v) have recently been published (Manning et al., 2020). The vaccine is composed of four salivary peptides from *A. gambiae* salivary glands that are common in many other mosquito vectors. The aim of the vaccine is to provide prophylactic protection against various mosquito-transmitted diseases. In the study, adult participants were assigned to one of three treatment groups: AGS-v vaccine, AGS-s vaccine, and adjuvant, or placebo. Treatment was delivered via subcutaneous injection at day 0 and day 21, after which the participants were exposed to uninfected *A. aegypti* feeding at day 42. The vaccine candidate was considered safe for its use in humans. Furthermore, participants who received the vaccine in combination with adjuvant mounted increased vaccine-specific IgG antibodies and cellular responses. All together, the results from this study suggest that AGS-v is an achievable option to implement as a vector-targeted vaccine.

## Conclusion

Arbovirus infections are a worldwide public health problem. To date, attempts to control the transmission of infection and disease occurrence in vulnerable areas have not been completely successful. Thus, new approaches are needed. Viral entry into the host takes place in the epidermis and dermis of the skin and is mechanically and chemically assisted by the mosquito. The saliva of the vector has the capacity to disturb both innate and adaptive immune responses. Various studies have reported the capacity of saliva components to drive a shift from Th1 (effective, desirable) to Th2 responses. Such a switch is achieved by altering cytokine, chemokine, and interferon production by the cells. Specific salivary compounds have also been reported to induce autophagy, as well as inhibit T and B lymphocyte proliferation and induce apoptosis. Overall, the added effects of these alterations of the immune response lead to enhanced viral replication, disease severity, and ultimately, transmission. Nevertheless, the current state of the art for salivary vaccine development is exciting, as an increasing number of animal studies are showing favorable results and human clinical trials for universal vector vaccines are already in the pipeline.

### Required Future Research to Fill Current Knowledge Gaps

Although there has been much research on the composition of mosquito saliva, complete functional proteomic profiles are not yet available. A significant number of studies are still based on transcriptomic or genomic approaches, which, although powerful, lack the capacity to determine 3D protein structure, post-transcriptional modifications, and specific biological functions. In terms of the diversity of the types of compounds being studied, there are also opportunities to expand our current knowledge. Most studies have thus far focused on protein identification and have left the metabolome and miRNA relatively unexplored. Furthermore, although the differential expression of salivary proteins in infected vs. uninfected mosquitoes has been studied (Zhang et al., 2013; Chisenhall et al., 2014), a complete differential proteome/metabolome/miRNA profile is not yet available. In addition, the mechanisms by which the virus affect the composition and abundance of salivary compounds in the vector are not well-understood. Studies on mosquito salivary factors and their effect on immune responses have been carried out using *A. aegypti* saliva. Other important vectors for arboviruses, such as *A. albopictus* and *Culex* ssp., are under-investigated. Thus, there are few comparative analyses of the various effects of saliva on the immune response between different vectors. The same is true for the differential salivary immune-modulatory effects of wildtype mosquitoes relative to those of laboratory-reared mosquitoes. Much has also been accomplished in understanding the host immune response to saliva components, after decades of research, which has set the foundation for the scientific research of recent years. Nevertheless, more extensive characterization of the effector cells involved in the response to mosquito saliva, along with their chemokine and cytokine signatures, is still needed. More complete knowledge of the interactions between saliva and primary host immune cells will make it possible to identify key cell populations/molecules/pathways that control the efficiency of infection. The same is true for the mechanisms by which saliva components reshape the local immune response at the bite site, as a more profound understanding of these mechanisms can be used in the development of treatment.

## Author Contributions

DG contributed to the writing and editing of the review. DM and TC contributed to the final form of the manuscript and its improvement. DG and DM contributed to designing the table and figure. All authors contributed to the idea and form of the manuscript and approved the submitted version.

## Conflict of Interest

The authors declare that the research was conducted in the absence of any commercial or financial relationships that could be construed as a potential conflict of interest.
